# Clinical potential of sensory neurites in the heart and their role in decision-making

**DOI:** 10.3389/fnins.2023.1308232

**Published:** 2024-02-13

**Authors:** Mugdha Tendulkar, Reshma Tendulkar, Parmdeep Singh Dhanda, Alpa Yadav, Mukul Jain, Prashant Kaushik

**Affiliations:** ^1^K. J. Somaiya Medical College and Research Centre, Mumbai, India; ^2^Vivekanand Education Society’s College of Pharmacy, Mumbai, India; ^3^Department of Biochemistry, Punjab Agricultural University, Ludhiana, India; ^4^Department of Botany, Indira Gandhi University, Rewari, India; ^5^Cell and Developmental Biology Lab, Center of Research for Development, Parul University, Vadodara, India; ^6^Department of Life Sciences, Parul Institute of Applied Sciences, Parul University, Vadodara, India; ^7^Chaudhary Charan Singh Haryana Agricultural University, Hisar, India

**Keywords:** sensory neurites, sensory transduction, decision-making, neural development, neurovascular unit

## Abstract

The process of decision-making is quite complex involving different aspects of logic, emotion, and intuition. The process of decision-making can be summarized as choosing the best alternative among a given plethora of options in order to achieve the desired outcome. This requires establishing numerous neural networks between various factors associated with the decision and creation of possible combinations and speculating their possible outcomes. In a nutshell, it is a highly coordinated process consuming the majority of the brain’s energy. It has been found that the heart comprises an intrinsic neural system that contributes not only to the decision-making process but also the short-term and long-term memory. There are approximately 40,000 cells present in the heart known as sensory neurites which play a vital role in memory transfer. The heart is quite a mysterious organ, which functions as a blood-pumping machine and an endocrine gland, as well as possesses a nervous system. There are multiple factors that affect this heart ecosystem, and they directly affect our decision-making capabilities. These interlinked relationships hint toward the sensory neurites which modulate cognition and mood regulation. This review article aims to provide deeper insights into the various roles played by sensory neurites in decision-making and other cognitive functions. The article highlights the pivotal role of sensory neurites in the numerous brain functions, and it also meticulously discusses the mechanisms through which they modulate their effects.

## 1 Introduction

The human body comprises an intricate network that is regulated by multiple mechanisms to ensure efficiency in its processes. There is a phenomenal coordination between different parts of the body when it comes to the function of decision-making. Past researchers state that the presence of two systems in the body influences the role of decision-making-analytic and experiential systems (Blomquist et al., 1987; Carlson et al., 1992). The existence of these systems supported the dual-system hypothesis formulated in the past. As per these theories, the experiential system is fast and considers emotional experiences and intuitions into consideration while making decisions (Ardell, 1994; Brodde and Zerkowski, 1994; Beissner et al., 2013). On the other hand, the analytic system is comparatively slow and takes into account logical reasoning while making decisions. These theories work well and adhere to the research of past decades from the arenas of interoceptive awareness and embodied cognition (Adolphs et al., 2019).

The human heart comprises an intrinsic nervous system that has the capacity to modulate short-term and long-term memory functions (Aru et al., 2012). It contains approximately 40,000 neurones, specifically sensory neurites. Sensory neurites are basically specialized nerve fibres throughout the heart which regulate cardiac activity. A network of such neurite ganglia comprises the intracardiac nervous system. It integrates the inputs of extrinsic innervation and plays a pivotal role in memory functions.

As per the theory proposed by Burr (2017), choice behavior is a consequence of deliberate commitments and logic which is unlike considering action and perception. Contemporary research on embodied decision-making explains the dissolved boundaries between cognition, action, and perception (Hopkins et al., 2000; Ciaunica et al., 2022). It also educates us on the strong interconnections between sensorimotor control which involves reciprocal interaction between sensorimotor and affective neural regions. Barrett and Barr in their studies effectively proved that the neural interactions associated with perception are representative of intricate associations between interoceptive information from the body and the exteroceptive cues in decision-making (Barrett and Bar, 2009; Adolfi et al., 2017). In a nutshell, decision-making is equally influenced by logical reasoning as well as intuition or so-called “heart instincts.” Emotional experiences play a vital role in reaching a decision along with contemporary logic (Barrett, 2017). The idea that the process of decision-making involves embodied cognition and somatic re-representation gives rise to an important conclusion. This embodied cognition originates from the embodied neural circuits further from the deep limbic structures to the frontal lobes of the cranial brain (Gatti et al., 1995; Cleeremans and Tallon-Baudry, 2022). This helps us reach the milestone that our neuroceptive processing heavily depends on the autonomic afferents (Farrell et al., 2001). There are two major neural systems interacting with these autonomic afferents, namely, the intrinsic cardiac network and the extrinsic cardiac network (Craig, 2002).

Decision-making is an important as well as inevitable aspect of human life varying from ascertaining insignificant to life-influencing decisions. The process of decision-making can be summarized as choosing the best alternative among a given plethora of options in order to achieve the desired outcome. This requires establishing numerous neural networks between various factors associated with the decision and creation of possible combinations and speculating their possible outcomes. In a nutshell, it is a highly coordinated process consuming a majority of the brain’s energy. This also supports the fact that making a lot of decisions also leads to fatigue as it is a metabolically expensive process. In case of ascertaining a decision, the sensory neurites modulate their effects by transmitting their neural signals through ascending pathways via the vagus nerve and spinal cord. These neural signals further travel to the amygdala, thalamus, hypothalamus, and finally to the cerebral cortex. The pre-established neural signals thus formed are remolded upon the intervention of the inputs of the intrinsic cardiac nervous system. Decision-making can be of various types such as physiological, behavioral, and psychological. The sensory neurites through their ascending pathways influence all three types of decisions in the cerebral cortex.

We can propose a novel framework for the outcome prediction based on the electrical activity signals of the heart. Moreover, heart rate variability (HRV) can also be employed for the comparison study as well as the autonomic nervous signals assessment tool. The evaluation model can be formulated based on the classification and regression problem solutions obtained from software such as CatBoost, Light BGM, and XGBoost (Klein, 2008). Upon considering the intricate organization of the autonomic system, there is not a single all-inclusive test that will accurately exhibit the function of a specific system in the human body. The comprehensive literature survey testifies the usage of numerous diagnostic tests that are based on the assessment of cardiovascular reflexes such as Valsalva maneuvre, deep breathing, cold pressor test, and isometric handgrip test (Mathias, 2003). Other diagnostic tests involve doing serial subtraction of a certain set of numbers known as the mental arithmetic test, orthostatic test, or examination of the haemodynamic responses as a consequence of active standing, head-up tilt test, and baroreflex sensitivity testing (Hilz and Dütsch, 2006). Moreover, the variabilities in the heart rate can be attributed to extreme emotions such as anxiety and euphoria; it can also be measured under different states of the heart such as emotional recognition as discussed before and the degree of cognitive impairment (Sinski et al., 2006). The measurement of neurotransmitters, particularly catecholamines circulating in the blood can also serve as a diagnostic tool for evaluating the autonomic nervous system (Zygmunt and Stanczyk, 2004). Recent studies have employed the usage of electrocardiogram (ECG) and skin sympathetic activity for predicting the outcome of intracerebral hemorrhage (Xing et al., 2023). Microneurography is yet another emerging diagnostic tool in which separate recordings of sympathetic nerve activity to the muscles (reflects the vasoconstrictor signal to the muscle) and sympathetic nerve activity to the skin vessels are employed. The quantitative nature of this tool also proves to be an added advantage for eliminating biases in the process.

The principal objective of this review is to deeply analyze the roles played by the sensory neurites in the heart and the neurocardiology associated with them. This forms the pivotal background across which we discuss the interactions between the sensory neurites and central neurones in order to maintain optimum cardiac output (Loewy and Spyer, 1990).

## 2 Neurocardiology: anatomical features

The cardiac nervous system consists of afferent neurones with their corresponding sensory neurites in the heart, efferent neurones that innervate the heart, and the neurones which aid the interaction between afferent and efferent neurones (Mittal and Lerman, 2002). Afferent sensory neurites are primarily located in the dorsal root ganglia, nodose, intrinsic cardiac, and intrathoracic extracardiac ganglia (Darvesh et al., 1987). The afferent neurites present in the dorsal root ganglia and nodose are responsible for pain and are somatic in nature. These lead to reflexes in autonomic tissues. These neurones tend to impact through sympathetic and parasympathetic efferent pre-ganglionic neural cells (central interconnecting neurones) which possess the tendency to synapse with efferent post-ganglionic neural cells (Coll et al., 2021). Intrathoracic feedback loops are formed by the interaction of intrathoracic cardiac afferent neurites with post-ganglionic efferent neurites (Barrett and Simmons, 2015). In order to weave a clear understanding of the neuroanatomy of these cells, we have thoroughly discussed the anatomy of these cells in the subsequent sections.

### 2.1 Cardiac afferent neurones

Experimental evidence suggests the presence of afferent neurones is not only limited to cardiac tissues in the dorsal root ganglia and node but also in the extracardiac, intrathoracic, and intrinsic cardiac ganglia (Zhuang et al., 2002). The sensory neurites associated with afferent neurones exhibit varied functions based on the locations in which they are present and are also influenced by the mechanical and chemical stimulus received by them (Kollai and Koizumi, 1979). According to the extensive studies carried out by Langley, it was found that sensory afferent neurites in the dorsal root ganglia and nodose are somatic in nature, whereas intrathoracic afferent ganglia are considered to be autonomic in nature (Langley, 1921).

### 2.2 Cardiac efferent neurones

Sympathetic and parasympathetic efferent post-ganglionic neurones are known to innervate coronary vessels and cardiac myocytes (Hirose et al., 2002). The two efferent limbs of the autonomic nervous system are known to regulate the heart in a reciprocal approach. In a nutshell, it suggests that when one limb activates, the other gets suppressed (Takahashi et al., 2006). This hypothesis representing reciprocal nature has been reiterated when the recent findings hint that both limbs can either be suppressed or activated simultaneously (Baiano et al., 2021b). Moreover, a few number of intrathoracic local circuit neurones acquire pre-ganglionic inputs from both the efferent limbs. The cumulated integrated end input of the efferent pre-ganglionic neurones and sympathetic/parasympathetic neurones include labyrinthine neuronal coordination at the heart level (Skok, 1973). In each region of the intrinsic cardiac nervous system, efferent post-ganglionic neurones are present which exert significant control over the discreet parts of the heart (Hadaya and Ardell, 2020).

### 2.3 Intrathoracic local circuit neurones

The intricate communication between different neurones occurs through the intrathoracic nervous system comprising local circuit neurones (Randall et al., 1996). Local circuit neurones primarily aid communication between neurones of different ganglions in the intrathoracic region. Some of these neurones have large diameters, whereas some possess relatively small diameters (Oh et al., 2006). The large diameter neurones consist of multiple nuclei. The collection of such neurones is called a rosette. Moreover, these rosettes are present in the extrathoracic cardiac region as well (Norris et al., 1977). The intrinsic cardiac nervous machinery also comprises neural cells that project their axonal regions into the neurones of other intrinsic cardiac ganglionic neurones (Pappone et al., 2004). These neural cells have the capability of imparting immunoresistivity toward specific peptide cells, for instance, vasoactive intestinal peptide and neuropeptide Y (Wiggers, 1914). These neurones play a major role in facilitating the interaction between various ganglions within the intrathoracic region.

### 2.4 Interactions of the peripheral autonomic neurones

It is a long-known fact that the intrathoracic ganglia are monosynaptic in nature. They distribute efferent parasympathetic (intrinsic cardiac ganglion) and sympathetic (extrinsic cardiac ganglion) centrifugal signals to the heart (Thompson et al., 2000). According to the research advances, it is clear to us that they also possess the ability to process centripetal signals (Yuan et al., 1993). This complicated neural hierarchy observed within this intrathoracic region is employed with the aid of inhibitory and excitatory synapses. Afferent neurones are known to receive communication signals not only from the cardiac sensory neurites but also from the pulmonary tissues and major thoracic vessels (Paton et al., 2005). It is also known that they receive signals from the spinal cord neurones as well. These spinal cord neural cells are believed to be indirectly impacted by the sensory neurites located elsewhere in the human body such as mechano-sensory neurites in the carotid arteries (Howell, 1915). While we do not possess sufficient information, an approximate organization of the intrathoracic cardiac neural system is proposed. This idea explains that these cardiac-afferent neurones influence the local circuit neurones which inadvertently alter autonomic efferent post-ganglionic neurones through the operation of various feedback loops. These intrathoracic cardiac reflexes heavily influence the cardiac rhythms on a beat basis even though these are practically disconnected from the centrally located neurones. Numerous chemicals are known to alter these extracardiac and intracardiac neurones such as acetylcholine, α-and β-adrenoceptor agonists, nitric acid donors, muscarinic agonists, purinergic agents, and hydroxyl radicals (Kuntz, 1934). In a nutshell, these intrathoracic cardiac reflexes render a long-lasting impact on the cardiac force as well as rate.

## 3 Cardiac control

The neuronic cells associated with heart synchronization are primarily situated from the insular cortex up to the heart. The role of intrathoracic cardiac ganglia in this neuronal hierarchy is considered to act as an efficient efferent relay point. As discussed before, intrathoracic ganglia are considered efferent checkpoints which are monosynaptic, that regulate the centrifugal signals to the heart (Huang et al., 1994). These sensory signals received by the afferents in the dorsal root ganglia and nodose are further processed by the central nervous system (CNS). After this processing of information, it significantly impacts the sympathetic post-ganglionic nerve cells in the paravertebral ganglia through its central nervous part. The parasympathetic post-ganglionic nerve cells in the target organ ganglia are known to process signals from medullary neural cells (Critchley and Harrison, 2013). It is a well-known fact that two branches of motor nerve cells tend to work in a complementary method.

Intrinsic efferent post-ganglionic cholinergic nerve cells derive synaptic signals from medullary pre-ganglionic neurones. These cholinergic efferent neurones are relatively fewer in number as compared to all the neurones present in the intrinsic cardiac nervous system (Cohn, 1990; Al et al., 2021a). Sympathetic efferent post-ganglionic neurones associated with heart regulation receive sensory signals from the cranial thoracic spinal cord pre-ganglionic neural cells. When these sympathetic efferent neurones are maximally activated, they increase cardiac dromotropism, inotropism, and chronotropism along with a significant decrease in the left ventricular end-diastolic volume (Dickerson et al., 1998). Reciprocally, maximal activation of parasympathetic efferent neurones leads to the suppression of inotropism (Bridges et al., 2007; Candia-Rivera et al., 2022). As per the research evidence of the recent era, it is known that the neurones of the intrathoracic ganglia including those present in the heart are in incessant communication with each other (Cooke, 1989; Al et al., 2020). As a result, numerous intrathoracic ganglionic neurones interacting with each other and the central neural system give rise to transient dependant reflexes that possess control over overlapping cardiac parts.

### 3.1 Intrinsic cardiac control

The late 19th century witnessed the discovery of the laws of the vertebrate heart researched from the quarantined cold-blooded vertebrate hearts. These extensive studies were then carried further by Patterson, Piper, and Starling to isolate the heart of the mammals. This idea explains that the extent of cardiac output directly depends on the amount of cardiomyocyte diastolic stretch, which is defined as the Frank–Starling hypothesis. The researchers highlighted the idea that the stroke volume of the ventricles directly depends on the regional cardiac muscle expansion during diastole (Craig, 2009). This measuring efficiency is ascertained by the degree of the chamber of returning venous blood. In the case of an explanted heart, when the chamber receives an increased flow of venous blood, it consequently leads to greater muscle stretch during diastole in such a manner that with each subsequent contraction, there is an observed increasing contraction leading to higher blood flow to the right/left ventricle.

### 3.2 Extrinsic cardiac control

The cardiomyocyte behavior is also controlled by the chemicals and hormones circulating in the heart which are directly released into the bloodstream by the nerve terminals of autonomic efferent post-ganglionic nerve cells (Brown, 1967; Avery et al., 2014). It would be worth mentioning here that the heart is enclosed within a pericardial sac that prevents organ displacement along with restriction of beat-to-beat ventricular diastolic distension (Cheng et al., 1997; Boehme et al., 2019). This anatomical protective feature nullifies the *in situ* distension during short-term ventricular diastole (Craig, 2003). It would be relevant to discuss the effect of extrinsic factors while considering the variance in cardiac output based on the changing body requirements in numerous physiological states.

### 3.3 Reciprocal thesis of cardiac control

The CNS is known to exert phenomenal reciprocal command over the cardiac indices through its two major branches. This process happens under the sole direct control of the central neural system, regulating parasympathetic (cholinergic) and sympathetic (adrenergic) efferent postganglionic nervous attributes (Chiou et al., 1997). An observed significant increase in the sympathetic efferent neuronal tone leads to increased cardiac inotropism, dromotropism, and chronotropism. In contrast, as per the reciprocal hypothesis, the reverse occurs when the parasympathetic (medullary) efferent pre-ganglionic nerve cells are activated (Heberdeen, 1772). As a result, the intrinsic cardiac ganglia (only parasympathetic) and intrathoracic extracardiac neurones (only sympathetic) tend to act as relay checkpoints that function reciprocally. When efferent parasympathetic (inhibitory) motor neurones become highly activated, the process regulated by the sympathetic (augmentor) motor neurones gets suppressed and vice versa.

## 4 Transduction

The diverse interlinked cardiac regions, the major cervical and intrathoracic vessels along with coronary musculature are incessantly transduced by chemosensory or mechano-sensory afferent nerve cells (Dusi and Ardell, 2020). These signals are then received by the secondary neurones which are located spatially throughout the cardiac neuronal hierarchy. The process of transduction basically aims at transferring signals and helping them interact with the cell receptors of the target organs in order to elicit a desired response. Research evidence hints that the restricted group of cardiac afferents primarily transduce chemosensory or mechano-sensory information (McAllen and Spyer, 1976). The mechano-sensory afferent nerve cells are those that possess the capability of transducing mechanical deformation in the parts of neurites. Numerous afferents convert multimodal stimuli along with detecting mechanical and chemical modifications simultaneously. Cardiac afferent neuronal stomata are dispersed throughout the caudal cervical, dorsal root ganglia of cranial thoracic origin, nodose root ganglia, and cardiac intrathoracic ganglia. These sensory neurites lie highly compacted near the regions of inferior and superior vena cava, dorsal atria, outflow passages of ventricular musculature, inner aortic arch, and sinoatrial (SA) node.

**Figure 1:**
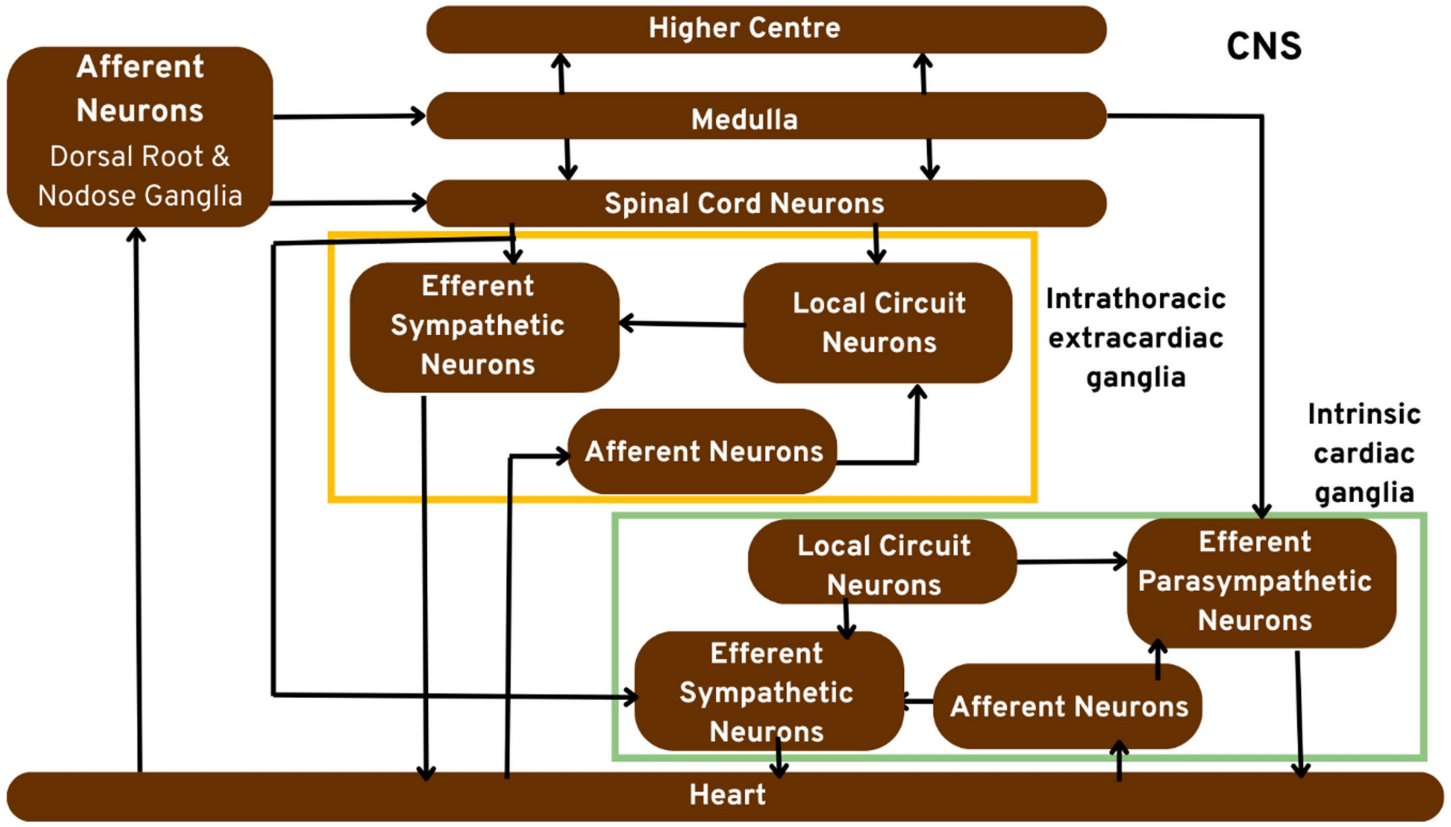
Pictorial representation of the cardiac neuronal hierarchy emphasising on the peripheral nerve components. Sensory cardiac signals are transduced by afferent nerve stomata in intrathoracic extrinsic and intrinsic cardiac ganglion through intrathoracic local circuit nerve cells to cardiac motor nerve cells.

Figure 1 portrays the hypothetical model of the cardiac neuronal hierarchy in great detail.

### 4.1 Chemosensory transduction

Diverse dorsal root ganglia and nodose afferent nerve cells in the heart aid in the transduction of the cardiac chemical environment. Their rate of exhibiting activity is aperiodic in nature, that is, 0.1–1 Hertz (Hz). These rates determine the relatively slow variance in the cardiac chemical conditions. Their activity skyrockets when the patient is suffering from hypoxemia. The cardiac chemosensory nerve cells are adept at transducing a wide array of chemicals individually. The modifications in the activity of these neurites indicate the comparable concentration of individual chemical which is ultimately adept of transduction. In short, even a single afferent nerve cell possesses the ability to generate numerous activity sequences in the power spectra when its associated sensory neurites come in contact with a plethora of chemicals (Bethesda et al., 2000). This occurs when one afferent nerve cell can concurrently transform into second-order nerve cells concerning novel signals of many chemicals. These chemicals discussed here are basically those released from the ischium myocardium in increasing amounts (Allen and Friston, 2018). This whole process ultimately increases the efficiency of signal transduction of multiple chemicals to second-order neurones.

### 4.2 Cardiac mechano-sensory transduction

A highly restricted community of cardiac nerve cells transduces phase-based mechanical alterations such that the associated neurites go through it every cardiac cycle. In the physiologic conditions, its phasic attributes are heavily dependent on the locations of their associated sensory neurites, indicative of muscle deformation locally for every cardiac cycle (Chechetto, 2004). An instance of their novel mechano-sensory transduction is exhibited by the mechano-sensory neurites present in the ventricular papillary muscle capable of transducing muscle deformation locally in an approximately linear method. It is previously known that these papillary muscles generally stretch when its chordae tendinae is exposed to increasing amounts of loads (Ali et al., 1990). Due to this, the muscle tends to undergo length regression when pressure increases in the right ventricular systolic order to instigate their activity reduction. When this right ventricular systolic pressure decreases due to less amount of blood coming from the atria, the right ventricular papillary muscle adjuncts itself to overcome the decreased tension on its chordae tendinae by increasing its length (Stoyek et al., 2021). This leads to an increase in their activity.

The outflow branches of the left and right ventricles mechano-sensory nerve cells transduce deformation locally in an exponential, positive way (Butler et al., 1990). Their behavior is observed to be at a peak during the generation of maximum chamber pressure. The left ventricular mechano-sensory nerve cells exhibit a plethora of intricate activity sequences as a consequence of increased systolic pressure. In essence, these ventricular mechano-sensory neurones refrain from transducing ventricular dynamics in an algebraic method as compared to peak chamber pressure generation (Babo-Rebelo et al., 2016). Some ventricular mechano-sensory neurones tend to transform diastole highlighting the fact that these nerve cells regulate a wide array of ventricular dynamics.

### 4.3 Afferent neuronal transduction

There is sufficient research evidence suggesting that a smaller number of afferent neurones either purely transduce chemosensory or mechano-sensory information. Mechano-sensory afferent nerve cells are those with the ability to transduce contortion in the regions where their cardiac neurites are located (Bosnjak and Kampine, 1989). The chemosensory nerve cells generally transduce modification in the chemical environment in the regions where their sensory neurites are placed. Numerous afferent neurones transduce multimodal stimuli and detect chemical and mechano-sensory modifications concurrently. The anatomical features indicate that these cardiac afferents are located throughout the caudal, cervical, nodose ganglia, and cranial dorsal root ganglia (Azevedo et al., 2016).

### 4.4 Intrathoracic vascular mechano-sensory transduction

Anatomical explorations suggest that a large population of these afferents is located in the dorsal root ganglia, nodose, and extracardiac (Baiano et al., 2021a). Intrathoracic ganglia have their associated cardiac neurites present in the adventitia of major vessels in the intrathoracic region. They are seen particularly in the bases of the vena cava and alongside the inner arch of the thoracic aorta (Candia-Rivera et al., 2021). They are observed to only transduce with the neurones belonging to the same ganglionated plexus. This highlights the capability of nerve cells in one intrathoracic locus to generate control over greatly divergent ventricular and atrial regions (Frank, 1959).

### 4.5 Heart–brain interactions and hierarchical control

**Figure 2:**
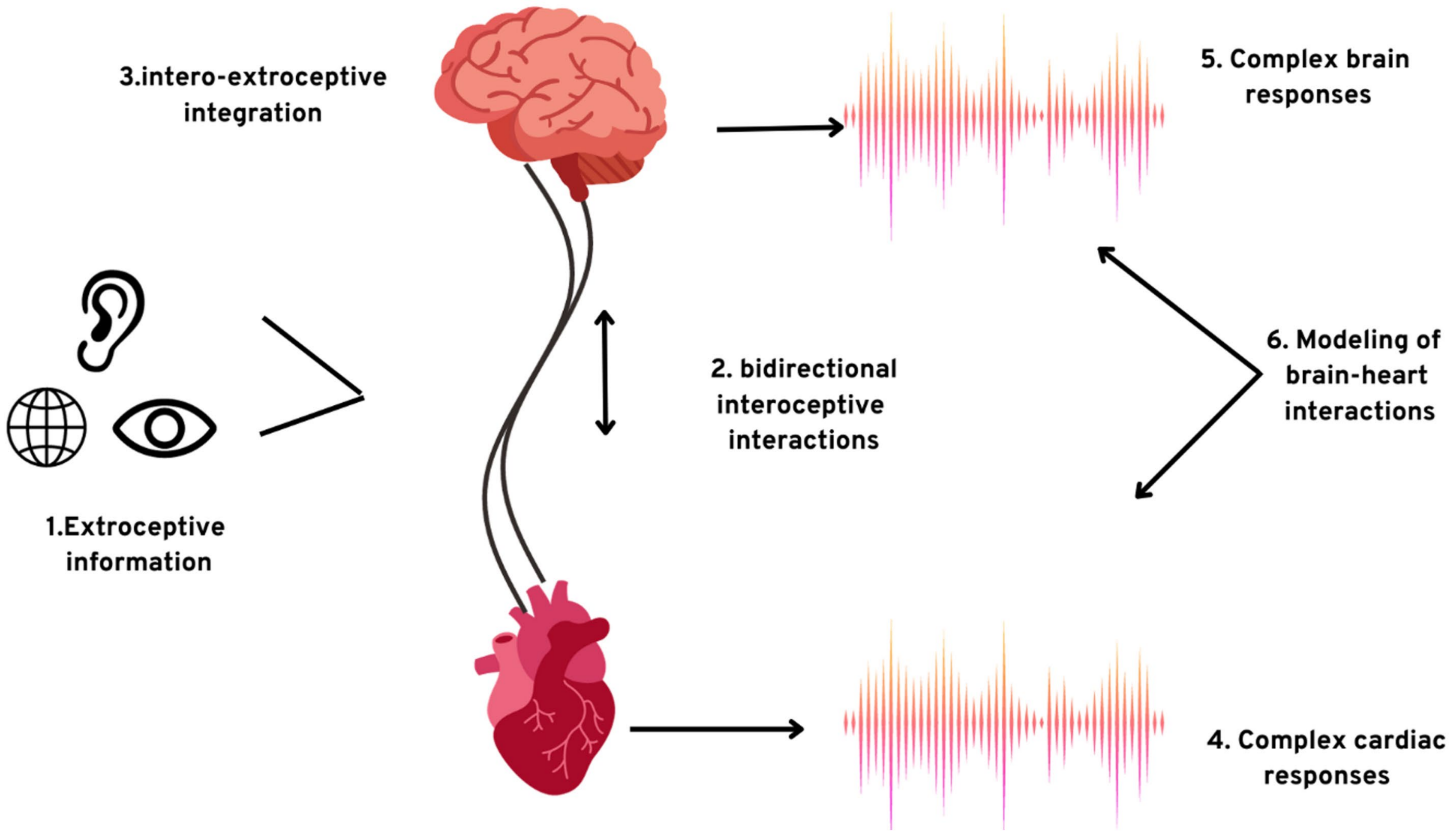
The exteroceptive–interoceptive integration. (1) Information processing from exteroceptive inputs occurs concurrently with; (2) two-way communication among the interoceptive channels and the brain; (3) integration of signals from the brain received from the sources; the results of the integrations are (4) intricate autonomic responses and (5) complex brain responses; (6) consciousness can also be studied by establishing the model plausible causal integration among the brain dynamics and intricate cardiac activity.

The sensory information generated from the major vessels and the heart instigates peripheral and central reflexes, thus dominating the cardiovascular motor nerve cells. These cardiac motor neurones have their cell bodies embedded in the brainstem of the human brain (Gebber et al., 1996). Figure 2 portrays the crosstalk of the brain and the heart which will help us gain greater insights into the close integration of the brain and the heart. This domination is simplified into two foundational problems:

- Discussion of how the afferents transduce the cardiac environment indirectly or directly to cardiac motor nerve cells.
- The latency of reflexes and type of signals transform these nerve cells.

It is observed that the neurones in the intrathoracic region are highly responsive to inhibitory or excitatory amino acids (Hanna et al., 2020). These reflexes comprise inhibitory/excitatory synapses. The observed variance in their latencies is highly dependent on the separation of afferent stomata to the target organ along with the number of synapses involved (Horackova and Armour, 1997). The event of directly transducing cardiovascular mechanical signals to cardiac motor nerve cells indicates an unsteady control condition. This comparable slow transduction through local circuit nerve cells to cardiac motor nerve cells of the chemical environment in numerous cardiac cycles aids in long-term control.

#### 4.5.1 Mechano-sensory vascular cardiac reflexes

The cardiac-arterial reflexes are known to be generated by the mechano-sensory neurites in the intrathoracic aorta and carotid arteries (Yuan et al., 1994). There are specific mechano-sensory neurites linked with the specific afferents which are seen to be compacted in the adventitia of the inner arch of the intrathoracic aorta and carotid bulbs. These neurites transduce arterial wall deformation through their nodose ganglia stomata to the nerve cells in the nucleus of the solitary branch. This initiates medulla-rooted short-latency activation of the parasympathetic efferent cardiac pre-ganglionic nerve cells (Wilbert and Morton, 2002). These facts support that numerous cardiac vagal afferents exhibit phase-based activity showcasing arterial events. This influences parasympathetic efferent cardiac neuronal dominance of the SA node distinctively through every cardiac cycle. This leads to the initiation of short-lived variance in the heart rate.

#### 4.5.2 Mechnosensory and chemosensory cardio-cardiac reflexes

The brief scaling attributes of the rapid-retorting cardiac mechano-sensory nerve cells initiate short-latency reflexes. These reflexes are in control of a specific large number of cardiac motor nerve cells (Murphy et al., 1994). Their sufficiently short separation of the first synapse enables distinctive excitation of the motor nerve cells during particular periods of the cardiac cycles. This exerts beat-to-beat integration of regional contractility and heart rate. These mechano-sensory reflexes are primarily under neuronal control (Hirsch et al., 1999).

The aftereffects of the exposure of these sensory neurites of specific chemosensory neurites to a greater number of chemicals can reconfigure the linked activity for greater periods of time followed by the dismissing of that chemical signal (Huang et al., 1996). This highlights neuronal memory. They are present in large numbers which allows them to influence greater populations of cardiac nerve cells over uncountable cardiac cycles.

#### 4.5.3 Neuronal memory

Neuronal memory can be simplified as a property exhibited by the large number of interactive nerve cells in an organismic species similar to their previous experiences that influence their present condition. A hypothesis suggests that the interposition of a population of nerve cells that cumulatively exhibits memory ensures steady dominance over cardiac motor function in the backdrop of modified heart conditions (Furukawa et al., 1990). The intricate integration and coordination of regional dynamics and heart rate is highly dependent on the mechano-sensory transduction comprising little memory, unequivocally short-latency reflex based. The cardiac chemosensory nerve cells exhibit working memory indicative of the local cardiac chemical environment (Al et al., 2021b). It operates in such a way that once the stimulus is taken away, behavioral alteration recurrently continues. This displays unhindered transduction of informative signals to the second-order neurones. The cardiac signals generated during one cardiac cycle can be further saved by the large populations of the intrathoracic local circuit nerve cells through integrated linkages to ensure dominance over cardiovascular nerve cells during cardiac cycles. There exists a short-lived memory retention in intrathoracic nerve cells which are practically disconnected from the central ones (Crucianelli et al., 2022). This displays that this capability is possessed solely among the intrathoracic neuronal hierarchy.

## 5 Role of neurites in brain functions

The sensory neurites carry out the important function of transmitting information to the brain and the spinal cord (collectively referred to as the central nervous system) from the heart. This tight-linked integration of the organs ensures point-to-point coordination (Barone et al., 1995; Adam et al., 2015). This function significantly affects our pivotal functions such as decision-making andcognition, and as per different circumstances, can lead to numerous diseases (Cummings et al., 2004; Blumenfeld, 2021). As described above, their potential for controlling signals via reflexes makes it possible for them to influence brain activities. This interesting discussion is thoroughly explained in the following sections.

### 5.1 Cognition and consciousness

Consciousness is basically the awareness of the self and the external environment. The monitoring of the visceral attributes aids in observing the adaptations to the alterations in the external conditions (Gurel et al., 2022). All the bodily processes carried out with the basic goal of ensuring optimal functioning of the body are referred to as homeostasis. There is another terminology involved known as allostasis, which primarily accounts for all the processes involved with a view to anticipating the future needs of the human body (Block, 2005). Incessant maintenance of allostasis requires cognitive functions, for instance, understanding, memorizing, learning, and perception (Cardinal et al., 2006). We can attribute these allostatic-homeostatic regulations to the alterations in the autonomic tone and cognitive functioning that have been considered in neuro-visceral interlinking models (Appelt et al., 2011). As per Damasio’s somatic marker hypothesis, the presentation of the bodily conditions in the brain mainly constitutes emotions, which heavily influence our approach to decision-making and aid in molding our morals and views (Babo-Rebelo et al., 2019). Numerous pieces of experimental evidence on interoception are determined to elaborate on the involved mechanisms for the detection of bodily signals and their effects on the dynamics of the brain. The process of interoception involves the detection of gut, cardiac, and respiratory signaling. From a broader perspective, interoception includes the physiology of the whole human body comprising affective youth, thermosensation, and pain (Azzalini et al., 2021). The innumerable functions associated include allostatic-homeostatic regulations in addition to high- and low-order cognition. In this scenario, it is known to us that the brain has the tendency to make predictions based on prior interoceptive and exteroceptive data (Andresen et al., 2004). These interoceptive processes are capable of remolding exteroceptive and metacognitive awareness. Many hypotheses have been proposed stating that bodily conditions are designated as a central factor in order to build your own self (Burwash et al., 1993; Banellis and Cruse, 2020). In this, the neural monitoring of ascending visceral inputs would be influenced by the subject activity. These hypotheses ultimately highlight the fact that the regulation of the body is not separated from cognition. The theoretical evidence greatly supports the influence of cardiac activities on cognition and consciousness (Waldman et al., 2006). Contemporary research evidence suggests that our behavior corresponds to the cardiac cycle, hinting that the heart timings serve as inputs for brain optimization. Research suggests that increased perception is observed when the stimulus is presented as diastole as compared to systole (Randall et al., 2003). In addition, it also suggests that sensory processing is significantly enhanced during diastole (Cardinal, 2004). This phenomenon can be attributed to the enhanced sensory processing of the brain at diastole hinting that the baroreceptors-originated afferent signals generated at systole-attenuated cortical excitability (Chen et al., 2006; Scanavacca et al., 2006). Moreover, it has been observed that active data sampling, motor/reaction excitability, and visual search significantly improved when the stimulus is received during systole. These multiple clinical pieces of evidence suggest that the brain has the tendency to effectively optimize cognitive processes depending on the cardiac phase (Rubio et al., 1969; Cheng et al., 1999). Against this backdrop, there is a clear understanding that active action and sampling attention are improved during systole and passive perception in an enhanced state during cardiac diastole. Hence, we come to know that interoceptive signals greatly influence plasticity (Vance and Bowker, 1983). In this highly evolving era, the question of the relation between the cardiac cycle and the timing of action/perception is still unanswered.

### 5.2 Decision-making

Decision-making as discussed before can be enumerated into three types—physiological, behavioral, and psychological. The physiological decisions encompass decisions modulating the body’s physiological function such as the levels of cortisol and variance in heart rate. Behavioral decisions involve formulating an alternative that will further influence the behavior of an individual. This approach can be attributed when the same human being behaves differently according to the situation, location, and the surrounding people. Psychological decision-making is basically weighing rational thoughts and emotional aspects, reaching a final conclusion after considering all the factors. This is a more complex type of decision-making as it involves the rationality of facts and the irrationality of emotions.

**Figure 3:**
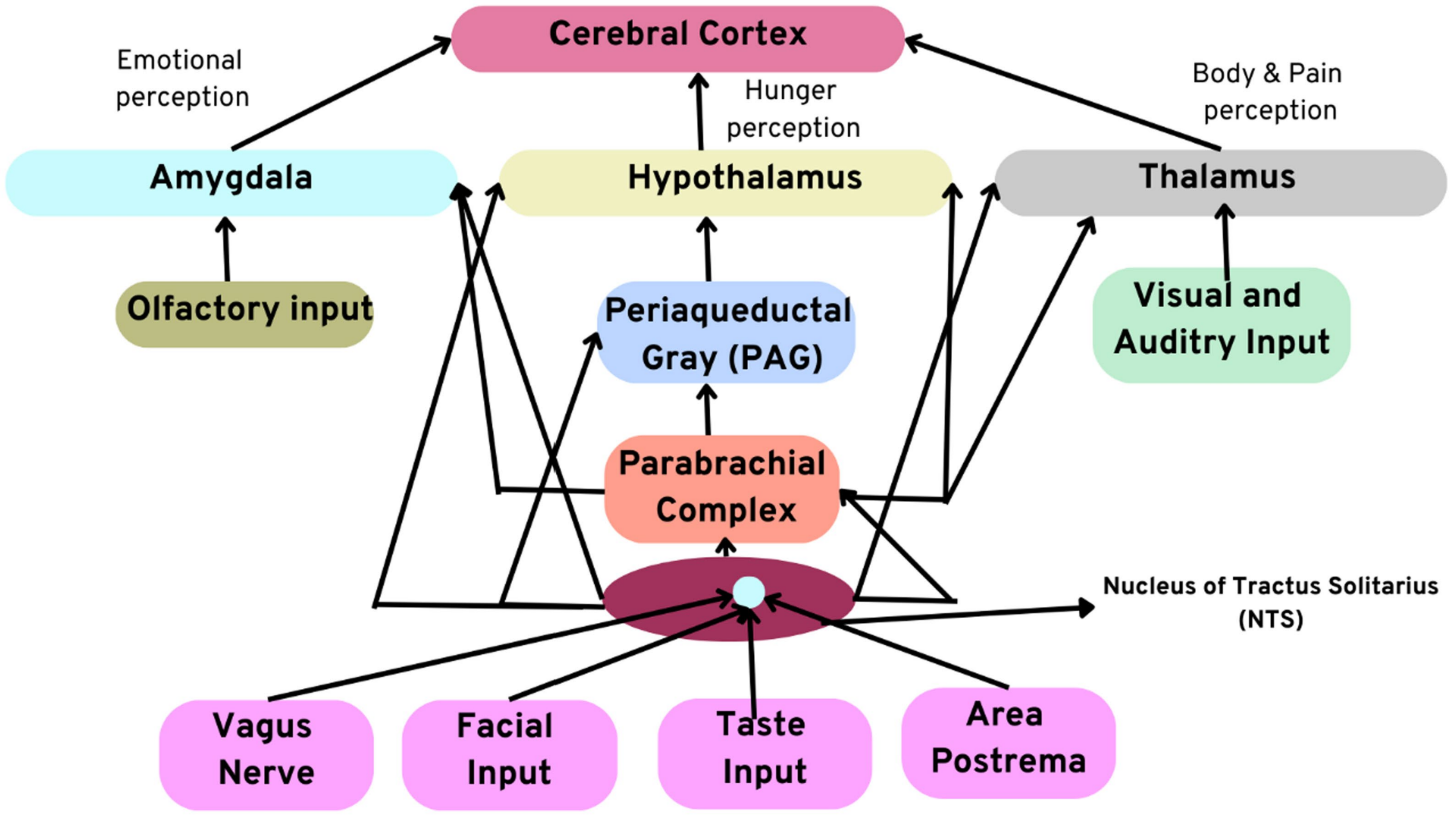
Illustration depicting afferent pathways through which the intrinsic cardiac neural system modulates its effects on the brain. Note the direct connections between the amygdala, thalamus, and hypothalamus.

Moreover, the heart also acts as a hormonal gland by secreting atrial natriuretic factor (ANF), atrial peptide, and atrial natriuretic peptide (ANP). An increase in the atrial peptide is known to significantly reduce stress levels and influence behavioral decisions and motivation in the brain. Recent research suggests that the heart also secretes and releases the neurotransmitter oxytocin responsible for the feelings of love and bonding. It influences psychological decision-making. It was also found that the concentrations of the neurotransmitter oxytocin are the same as the concentration in the brain, which suggests that the brain and the heart contribute equally to the emotions of love and social bonding. Figure 3 illustrates the numerous afferent pathways through which the sensory neurites in the heart modulate their effects on the brain.

The level of interoceptive awareness has been evaluated through numerous tasks of sensations associated with heartbeats. The insula in the cerebral cortex of the brain is primarily responsible for interoceptive awareness networks. The major role of the insula in perpetual awareness is backed by the experimental proofs observed from the neurodegeneration and its observed outcomes. Research evidence has proven the interrelationship between the insula and decision-making performance (Malliani, 1982; Patterson et al., 2005). A hypothesis has been proposed stating an intricate interrelationship between interoception and the interaction with the external stimulus. The neural responses generated by the heartbeats entitled as heartbeat-evoked responses (HERs) have been considered as a quantifiable unit for interoception (Levy and Martin, 1979; Scridon, 2022). These HERs are correlated with the functions of attention which can be either of interoceptive or exteroceptive origin. This observed functional association highlights that HERs can be used to evaluate as biomarkers of heart–brain interactions, particularly heart–brain communication (Murphy et al., 2000; Po et al., 2006). They are responsible for perpetual awareness. Prediction of perception in visual recognition points that neural responses to heartbeats play a pivotal role in molding conscious experience. This aids in imparting differential activation in cortical regions. HERs are also cognate with the perception of somatosensory origin in tactile recognition (Moravec and Moravec, 1987; Plecha et al., 1988). This influences our ability to make decisions. Thus, proving the capability of HERs in linking internal signals during conscious perception and detection of auditory malfunctions, exhibiting that the cortical potentials that are amalgamated to heartbeats portray the brain’s expectations of the external environment (Hillarp, 1960; Hanna et al., 2021).

Emotions and feelings are syndicated with the regulation of the attributes of the body. Innumerable cohort results have supported the fact that HER alteration in healthy controls, as against the variance in neurodegenerative disorders, is federated with advanced emotion regulation followed by a heartbeat sensation function (Huang et al., 1995; Kresh and Armour, 1997). This evidence highlights that HERs and the function of interoception jointly aid the process of priming emotional processes.

### 5.3 Clinical implications of sensory neurites

The ever-evolving scientific sphere has provided us with evidence that the intrinsic cardiac neural system modulates the cardiac pathological states. A high increase in cardiac sensory inputs basically observed in the case of regional ventricular ischaemia leads to unrestrained activation of cardiac second-order nerve cells in addition to the nerve cells in the neuronal hierarchy (Hopkins and Armour, 1984; Katchenova et al., 1996). To cite a few examples, receiving an avalanche of signals into the CNS influences cardiac neurones to a greater extent whose physiological response is being self-aware of the modified cardiac status (Forsgren et al., 1990; Horackova et al., 1996). Table 1 portrays the important clinically significant relationships between heart–brain and brain–heart.

**Table 1:** Clinically significant brain–heart and heart–brain relationships.

Brain–Heart	Heart–Brain
Ventricular arrthymia after stroke	Cardiogenic brain embolism
Reflex syncope	Cardiogenic syncope
Transient ischemic attacks before cardiac death	Bacterial endocarditis
Myocardial necrosis after stroke	Cardiac arrest and hypoxic–ischemic encephalopathy
Perioperative coronary death and carotid artery operation	Neurologic injuries and cardiac surgeries

Cardiac arrhythmias occur due to overactivation of the insular cortex in the brain. On the other hand, over-activation of specific elements within the intrinsic cardiac system leads to the emergence of ventricular or atrial arrhythmias (Horackova et al., 1999; Dell’Italia and Ardell, 2004). Experimental evidence suggested that the ablation of specific neuronal elements in restricted ganglionated plexus in the heart leads to long-term repression of atrial arrhythmias (Hopkins and Armour, 1989; Crucianelli et al., 2018). This can be attributed to the excessive vagus nerve input to the cardiac neural system-induced atrial fibrillation after programmed atrial stimulation.

The majority of the cardiac sensory neurites linked with dorsal root ganglion afferent nerve cells and nodose are known to transduce a chemical that is released in greater amounts by ischaemic myocardium called adenosine. Sylvén et al. put forth experimental evidence suggesting that the adenosine released can be responsible for cardiac pain (Sylv́en, 2004). In accordance with the above evidence, afferent nerve cells sensitive to dorsal root ganglion purine play a monumental part in the affliction of limb pain (Scherlag and Po, 2006). The pharmacology of all these processes proves to us the undeniable interlinking between sensory neurites and the CNS, specifically the brain (Schwartz et al., 1978). The emergence of arrhythmias can be attributed to the release of microliters of neurochemicals, namely, angiotensin II and β-andrenoceptor agonists (Paton et al., 2005). These neurochemicals are administered along with the specific populations of the cardiac neurites. The receptors of these neurochemicals influence cardiac motor neurones, which in turn affect cardiac activity (Patterson et al., 1914; Oppenheimer and Hopkins, 1994). The pharmacological strategies to combat these diseases are known to target particular elements of the neuroaxis that indirectly support the function of cardiomyocytes.

## 6 Conclusion and perspectives

The proposed heart–brain interactions primarily comprise of accumulation and amalgamation of signals, elucidation, and body control. A majority of these processes are intrinsic in cognitive and physiological basis ranging from allostatic, homaeostatic control to advanced-level cognitive tasks. The theoretical perspectives dealing with cardiodynamics put forth numerous mechanisms, but the glitch is that the ascertainment of whether heart–brain interactions are convoluted in the neurobiology of consciousness needs to be made clear. The evolution in our knowledge of communicative mechanisms and mutual regulatory pathways among visceral and central systems might make our understanding of the topic more certain.

The study’s outcomes hint at the possibility of considering the accumulation of memory engrams in the brain of the recipient in the case of heart transplants. This can be of possible interest to clinicians to assess the memory transfer and elucidate the possible mechanisms through which it expresses the memory in the recipient. The gist of this review is to precisely unravel the neurocardiology of the unknown populations of the heart. It discusses the mechanisms and the intricate processes through which it modulates and renders control over our brain and vice versa. Self-consciousness is one of the distinguishing features of the human body. The consequences of self-consciousness are the powers of decision-making and cognition that are responsible for shaping life and communicating with the external world. The sensory neurites play a pivotal role in modulating the cardiac activity. These cells also impart their influence in certain clinical cardiac disorders. The mechanisms involved in these diseases and their physiology have been thoroughly discussed. Assessing consciousness from the perspective of embodied cognition will broaden our perspective and will prove to be the prime research question in the field of neuroscience.

## Author contributions

MT: Conceptualization, Investigation, Methodology, Project administration, Writing – original draft. RT: Conceptualization, Investigation, Methodology, Project administration, Writing – original draft, Writing – review & editing. PD: Data curation, Formal analysis, Software, Supervision, Writing – review & editing. AY: Data curation, Investigation, Resources, Validation, Writing – review & editing. MJ: Conceptualization, Formal analysis, Investigation, Resources, Supervision, Visualization, Writing – original draft. PK: Funding acquisition, Investigation, Methodology, Project administration, Supervision, Validation, Visualization, Writing – review & editing.
